# Supplementary material for: Norepinephrine regulates calcium signals and fate of oligodendrocyte precursor cells in the mouse cerebral cortex
Source: Nat Commun. 2023 Dec 8;14:8122. doi: 10.1038/s41467-023-43920-w (PMC10709653; doi:10.1038/s41467-023-43920-w)
Supplement: Supplementary file 1 — Supplementary Information [file 41467_2023_43920_MOESM1_ESM.pdf]

Supplementary Information

Norepinephrine regulates calcium signals and fate of oligodendrocyte precursor cells in the cortex

Frederic Fiore[1, *], Khaleel Alhalaseh[1, *], Ram R. Dereddi[1, 2], Felipe Bodaleo Torres[1]
Ilknur Çoban[1, 2], Ali Harb[1], Amit Agarwal[1, 2, §]

1. The Chica and Heinz Schaller Research Group, Institute for Anatomy and Cell Biology, Heidelberg University, Heidelberg, Germany
2. Interdisciplinary Center for Neurosciences, Heidelberg University, Heidelberg, Germany

* These authors contributed equally
§ Lead contact

Correspondence:
Amit Agarwal, Ph.D.
Heidelberg University
Institute for Anatomy and Cell Biology
Im Neuenheimer Feld 307
69120 Heidelberg, Germany
Phone: +49 6221-54-6115

Email: amit.agarwal@uni-heidelberg.de

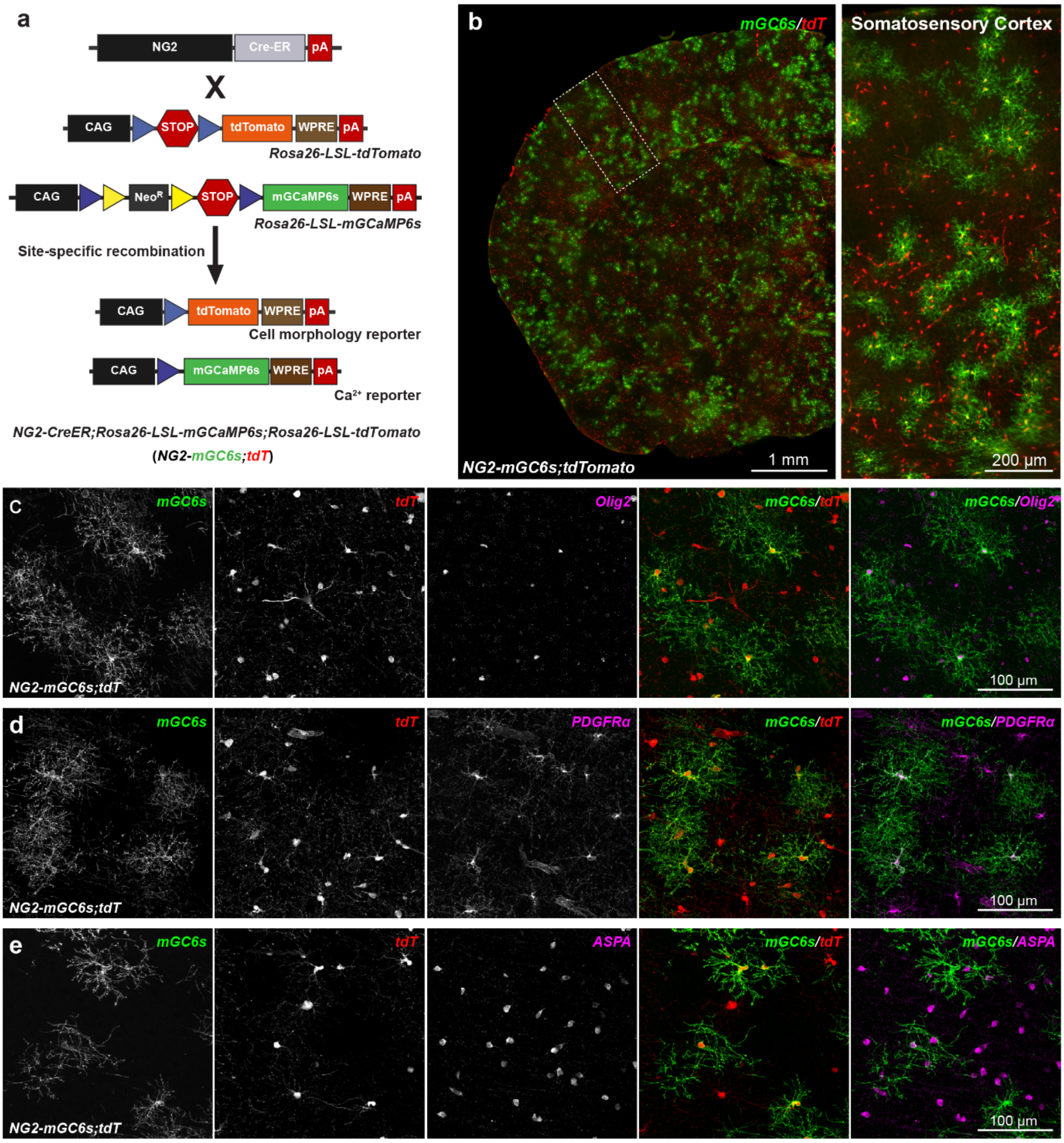

Supplementary Figure 1. Conditional expression of mGCaMP6s and tdTomato in oligodendrocyte lineage cells.

(**a**) Cartoon showing a triple transgenic strategy to express membrane anchored variant of GCaMP6s (mGCaMP6s) and tdTomato in OPCs using *NG2-CreER;Rosa26-LSL-mGCaMP6s;Rosa26-LSL-tdTomato* (*NG2-mGC6s;tdT*) mice. (**b**) (left) Coronal hemi-section of brain from *NG2-mGC6s;tdT* mouse stained for mGCaMP6s (mGC6s, green) and tdTomato (tdT, red). (right) Zoom-in image from the S1 cortex (boxed area shown in the left panel) showing oligodendrocyte lineage cells co-expressing mGC6s and tdT. Scale bars, 1 mm (left) and 200 µm (right). (**c**) Confocal microscopy images showing double recombined (mGC6s$^+$/tdT$^+$) cells expressing pan-oligodendrocyte lineage marker Olig2 (magenta). (**d**)

Images showing a subset mGC6s$^+$/tdT$^+$ cells were PDGFRα$^+$ OPCs (in magenta). (**e**) Images showing a subset of recombined mGC6s$^+$/tdT$^+$ cells expressing ASPA (magenta), a marker for mature oligodendrocytes. Scale bars, 100 μm (**c-e**).

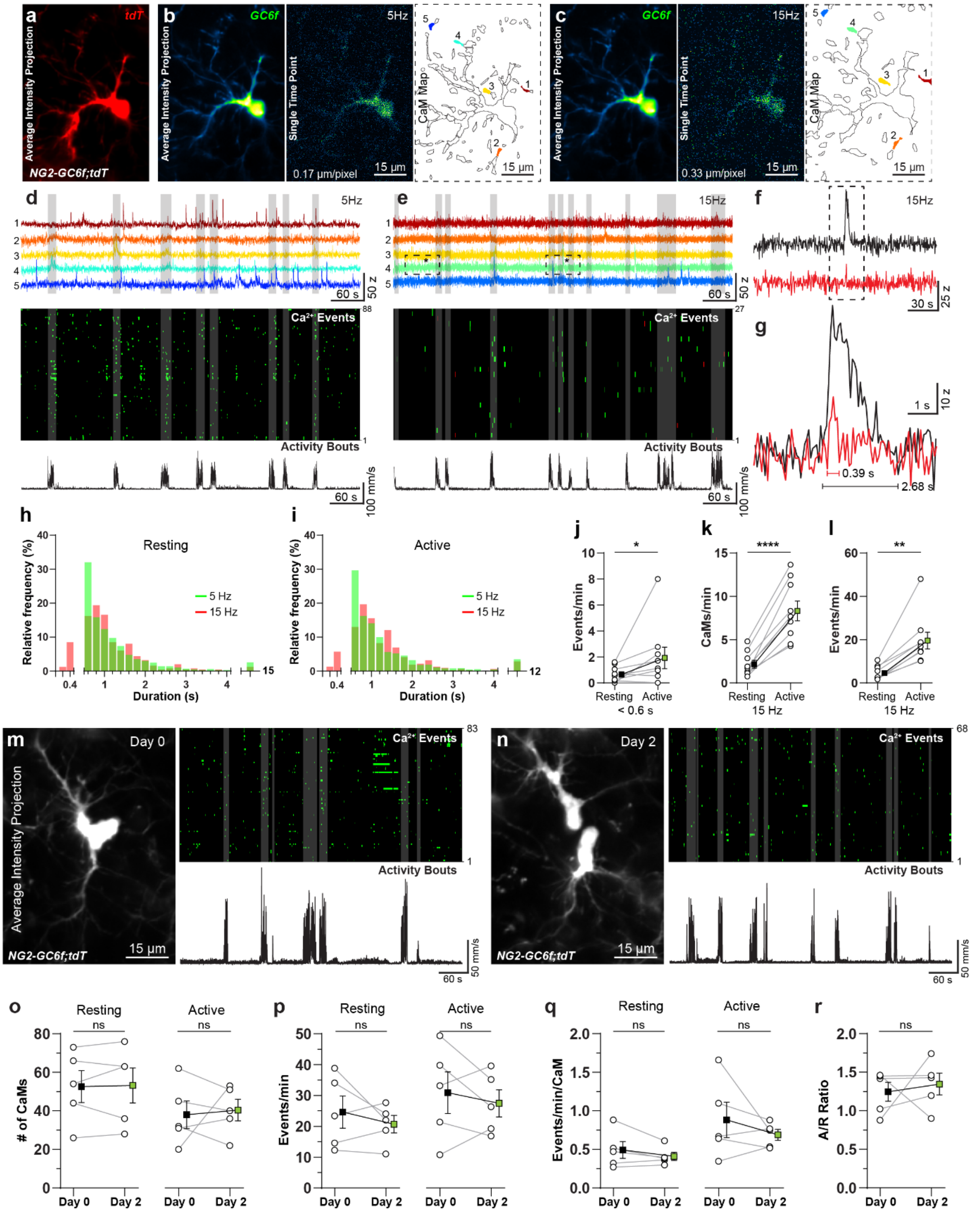

Supplementary Figure 2. Characterization of the fast Ca^{2+} transients in OPCs, and Ca^{2+} signal in the OPC undergoing cell division.

(**a**) Average intensity projection of an OPC expressing tdTomato in the S1 cortex. (**b-c**) Average intensity projection of GCaMP6f (left), a single time frame (center), and a map of the detected CaMs (right) during a 10-minute recording at 5 Hz (**b**) or 15 Hz (**c**). (**d, e**) Intensity vs time Ca^{2+} traces from 5 CaMs (top) at 5 Hz (**d**) and 15 Hz (**e**) corresponding to colors in **b**, **c.**

Raster plots show the temporal distribution of Ca^{2+} events at 5Hz (**d**) and 15 Hz (**e**), aligned with the mouse activity trace. Ca^{2+} events < 600ms are shown in red. Vertical grey bars indicate bouts of mouse locomotive activity. (**f**) Magnified Ca^{2+} traces from the boxed areas in **e**. (**g**) Zoom-in of fast (red) and slow (black) Ca^{2+} events detected at 15 Hz. (**h**, **i**) Histograms of the duration of Ca^{2+} events detected at 5 Hz (green) and 15 Hz (red) during resting (**h**) and active (**i**) phases. The first segment of the x-axis shows the distribution of Ca^{2+} events < 600ms. The last segment of the x-axis shows the distribution of Ca^{2+} events >4 s. (**j**) Graphs of the frequency of 'fast' Ca^{2+} events (<600ms). (**k**, **l**) Graphs showing the number of Ca^{2+} microdomains (**k**) and the frequency of Ca^{2+} events (**l**) during resting and active phases at 15 Hz. (**m-n**) (left) Average intensity projection of an OPC before (**m,** Day 0) and after (**n,** Day 2) cell division. (right, top). Raster plots show the Ca^{2+} events that occurred during Day 0 and Day 2, aligned with the mouse locomotion activity trace (right, bottom). Vertical grey bars represent bouts of locomotion. (**o-q**) Graphs of the number of active Ca^{2+} microdomains (**o**), the frequency of Ca^{2+} events (**p**) and the frequency of Ca^{2+} events per microdomain (**q**) during resting (left) and active (right) phases before and after cell division. (**r**) Graph showing the Active/Resting Ca^{2+} activity ratio (A/R ratio) before (Day 0, left) and after (Day 2, right) cell division. All data are presented as mean ± SEM. (**j-l**) N= 9 cells, N = 2 mice; Wilcoxon matched-pairs rank test: *P = 0.0391, **P = 0.0039. (**k**) Paired t-test: ****P < 0.0001. (**o-r**) n = 5 cells, N = 2 mice; Wilcoxon matched-pairs rank test: P > 0.05. Scale bars, 15 μm. Source data are provided in the Source Data file.

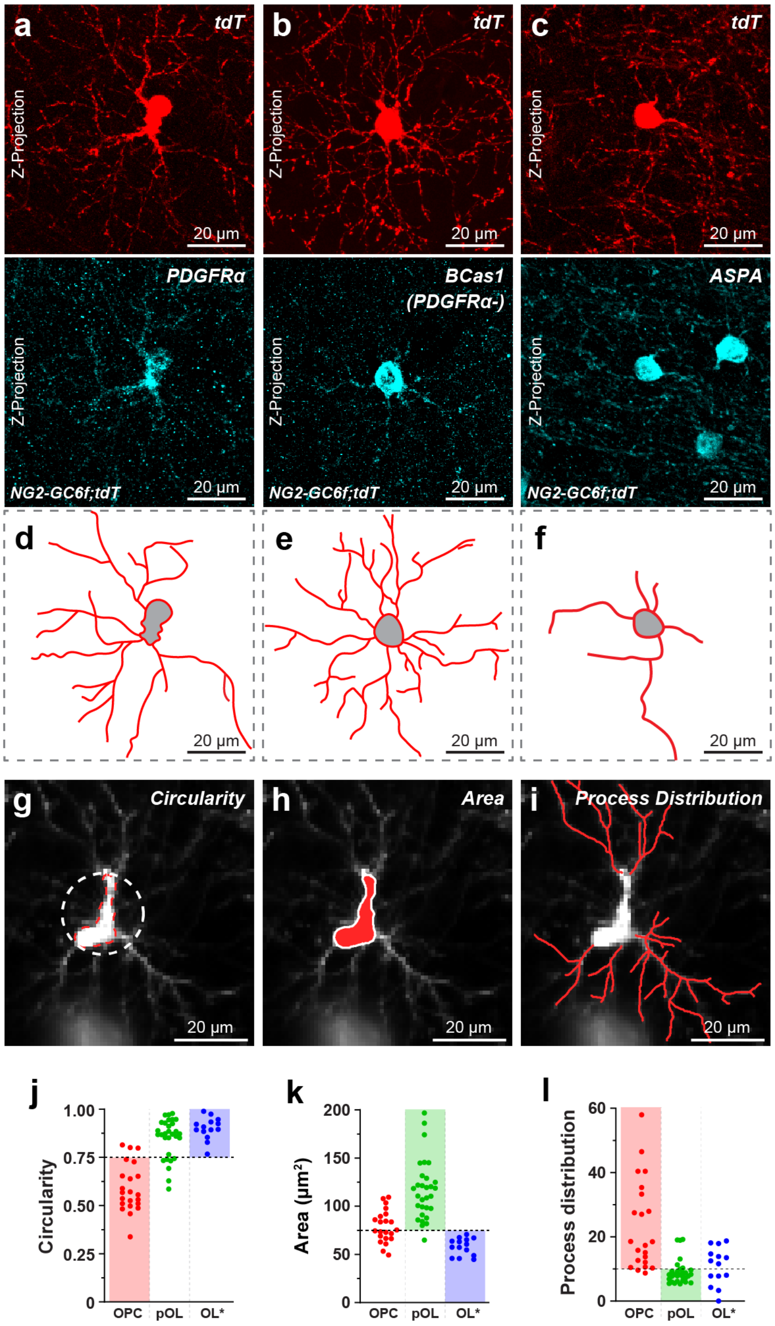

Supplementary Figure 3. Histological characterization of unique morphological features of distinct cell-types of oligodendrocyte lineage.

(**a-c**) (top) Max intensity projection of confocal z-stacks of cells expressing tdTomato, and labelled with anti-tdTomato antibody, in the S1 cortex of a *NG2-GC6f;tdT* mouse. (bottom) PDGFRα$^+$ OPC (**a**), BCas1$^+$/PDGFRα$^-$ pmOL (**b**) and ASPA$^+$ OL (**c**) each cell-type immunostained with respective markers. (**d-f**) Examples of the manual tracing of morphology of the cells shown in **a-c**. The processes are shown in red and soma is grey. (**g-i**) Schematic representation of the morphological criteria used to distinguish OPCs, pmOLs and OLs. (**j-l**) Scatter plots showing distribution of soma circularity (**j**), soma area (**k**) and process distribution

(**I**) of labeled OPCs, pmOLs and OLs. Horizontal dotted lines represent threshold values that most effectively segregate 2 or more populations of OLCs. Each cell-type is highlighted with a colored rectangle - OPCs, red; pOLs, green; OLs, blue). n = 66 cells; N = 4 mice. Scale bars, 20 µm. Source data are provided in the Source Data file.

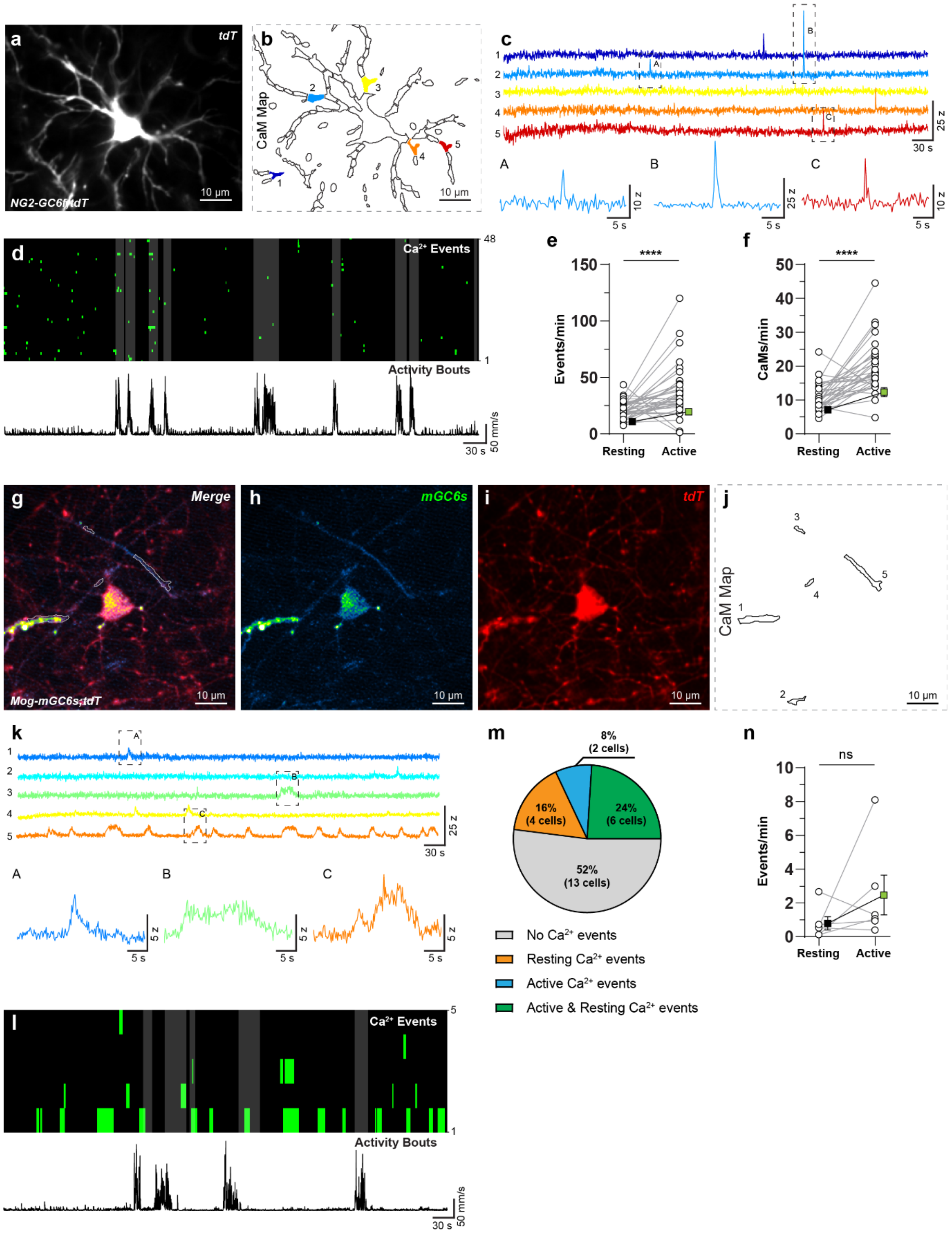

Supplementary Figure 4. Characteristics of Ca^{2+} signals in pmOLs and mature oligodendrocytes in awake mice.

(**a, b**) Average intensity time-series projection of tdTomato reporter expressed by a differentiating OLC (pmOL1) in the S1 cortex of *NG2-GC6f;tdT* mouse (**a**) and its associated CaM map (**b**). (**c**) (top) Intensity vs time Ca^{2+} traces from 5 CaMs corresponding to colors in

b. (bottom) Magnified Ca^{2+} signal peaks from the boxed areas (A, B, C). (**d**) Binarized raster plot showing detected Ca^{2+} events in all 48 CaMs (top) occurring during a 10-min imaging period, and mouse locomotion activity trace (bottom). Vertical grey bars in the raster plot represent bouts of locomotion. (**e**, **f**) Graphs comparing number of Ca^{2+} microdomains (**k**), and frequency of Ca^{2+} events (**l**) in pmOL1 during the resting and active locomotion phases. (**g-i**) A merged pseudocolored average intensity projections of time series image of a recombined OL (**g**) co-expressing membrane-anchored GCaMP6s (mGC6s, **h**) and cytosolic tdTomato (tdT, **i**) in *Mog-mGC6s;tdT* mice. The associated CaM map of OLs showing regions with active Ca_{2+} signals (**j**). (**k**) (top) Intensity vs time Ca^{2+} traces from 5 CaMs labeled in **j**. (bottom) Magnified Ca^{2+} signal peaks from the boxed areas (A, B, C). (**l**) Binarized raster plot showing Ca2+ signals in CaMs active during both resting and active phase during a 10-min imaging period (bottom). Vertical grey bars in the raster plot represent bouts of locomotion. (**m**) Pie chart showing the proportion of OLs that exhibited no Ca^{2+} events (grey), Ca^{2+} events only during resting phase (orange), Ca^{2+} events only during active phase (blue), and Ca^{2+} events during both active and resting Ca^{2+} phases (green). (**n**) Graph comparing number of events per minute of the active OLs. All data are presented as mean ± SEM. (**e-f**) n = 34 cells, N = 7 mice. Wilcoxon matched-pairs rank test: ****P < 0.0001. (**m, n**) n = 25 cells, N = 3 mice. Wilcoxon matched-pairs rank test: P > 0.05 (**n**). Scale bar, 10 μm. s, second. z, z-score. Source data are provided in the Source Data file.

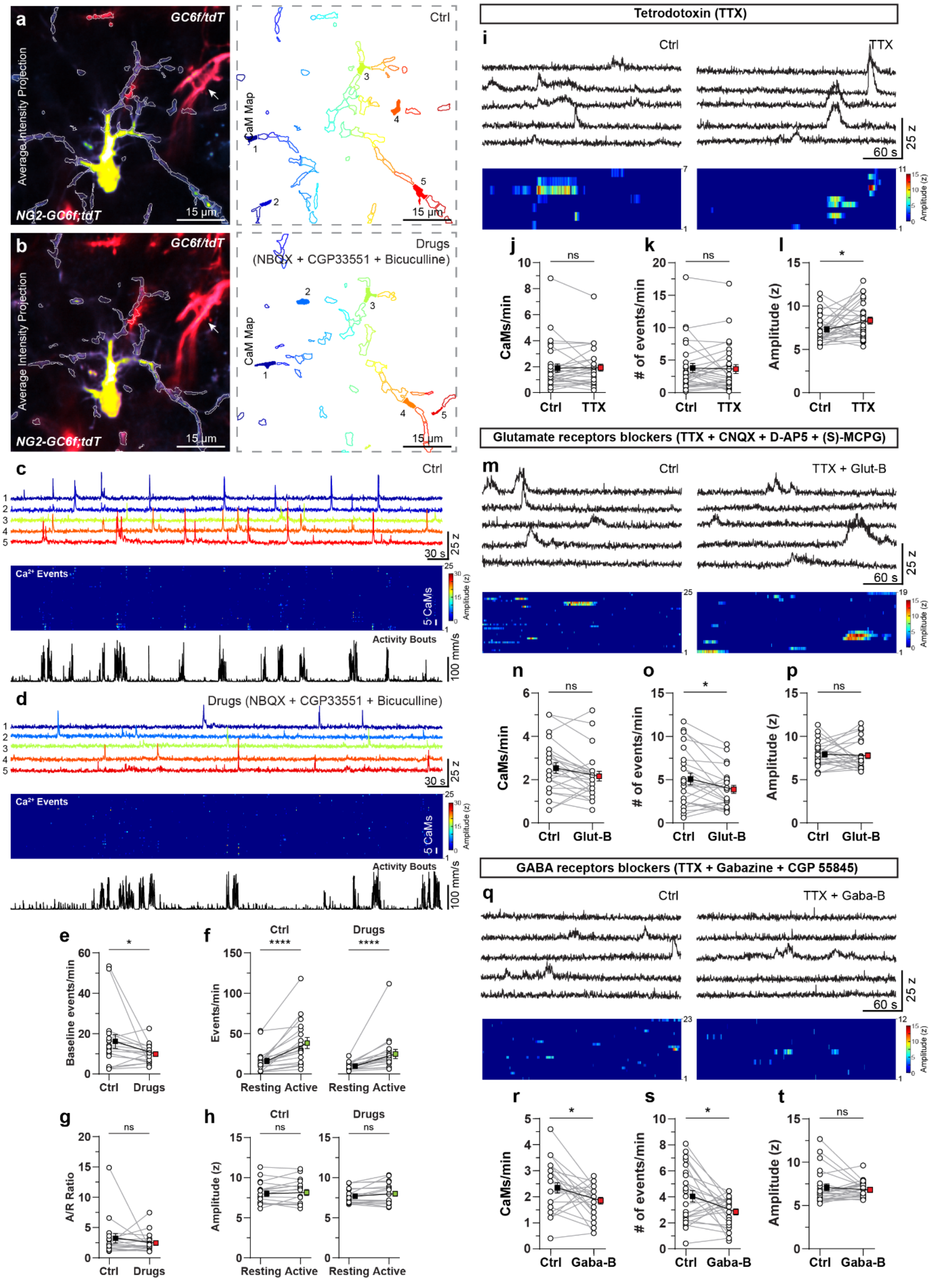

Supplementary Figure 5. Contribution of glutamatergic and GABAergic signaling to the spontaneous Ca^{2+} transients in OPCs *in vivo* and in acute brain slices.

(**a**-**b**) Average intensity projection of an OPC co-expressing cytosolic tdT (red) and GC6f (green) (left) overlaid with its associated CaM map (right) before (**a**) and after (**b**) i.p. injection of NBQX, CGP 33551 and Bicuculline. (**c, d**) Example traces showing Ca^{2+} signals in 5 CaMs (top) with a heat map of the intensity and temporal distribution of Ca^{2+} events (center), aligned with the mouse locomotion activity (black trace, bottom) before (**c**) and after (**d**) drug treatment. (**e**) Plots showing the frequency of resting Ca^{2+} events before and after the injection of the blockers. (**f**) Graph comparing frequency of Ca^{2+} events during resting and active phases, before (left) and after (right) the injection of the blockers. (**g**) Graph showing the A/R ratio in OPCs before and after the injection of the blockers. (**h**) Graph of the amplitude of Ca^{2+} events during resting and active phases, before (left) and after (right) the injection of the blockers. (**i**) Intensity vs time Ca^{2+} traces in Ctrl (top, left) and after TTX application (top, right). Heat-maps of the intensity and temporal distribution of Ca^{2+} events in the Ctrl (bottom, left) and after TTX application (bottom, right). (**j-l**) Graphs comparing number of active Ca^{2+} microdomains per minute (**j**), frequency of Ca^{2+} events (**k**), and the amplitude of Ca^{2+} events (**l**) in Ctrl conditions and after TTX application. (**m**) Intensity vs time Ca^{2+} traces in Ctrl (top, left) and after glutamate receptor blockers (Glut-B: TTX, CNQX, AP5 and MCPG) application (top, right). Heat-maps of the intensity and temporal distribution of Ca^{2+} events in the Ctrl (bottom, left) and after Glut-B application (bottom, right). (**n-p**) Graphs of the number of Ca^{2+} microdomains per minute (**n**), the frequency of Ca^{2+} events (**o**), and the amplitude of Ca^{2+} events (**p**) in Ctrl and after Glut-B application**.** (**q**) Intensity vs time Ca^{2+} traces in Ctrl (top, left) and after GABA receptor blockers (Gaba-B: TTX, CGP 55845 and Gabazine) application (top, right). Heat-maps of the intensity and temporal distribution of Ca^{2+} events in the Ctrl (bottom, left) and after Gaba-B application (bottom, right). (**r-t**) Graphs of the number of Ca^{2+} microdomains per minute (**r**), the frequency of Ca^{2+} events (**s**), and the amplitude of Ca^{2+} events (**t**) in control and after Gaba-B application. All data are presented as mean ± SEM. (**e-h**) n = 18 cells, N = 2 mice. Wilcoxon matched-pairs rank test: ****P < 0.0001, *P = 0.0385. (**j-l**) n = 29 cells, N = 6 mice. Wilcoxon matched-pairs rank test: P > 0.05 (**j**, **k**). Paired t-test: *P = 0.0365 (**l**). (**n-p**, **r-t**) 24 cells, N = 3 mice. Wilcoxon matched-pairs rank test: P > 0.05 (**n**, **t**). Paired t-test: *P = 0.0140 (**o**), *P = 0.0369 (**r**) and *P = 0.0182 (**s**). Scale bars, 20 μm. ns, not significant. s, seconds. z, z-score. Source data are provided in the Source Data file.

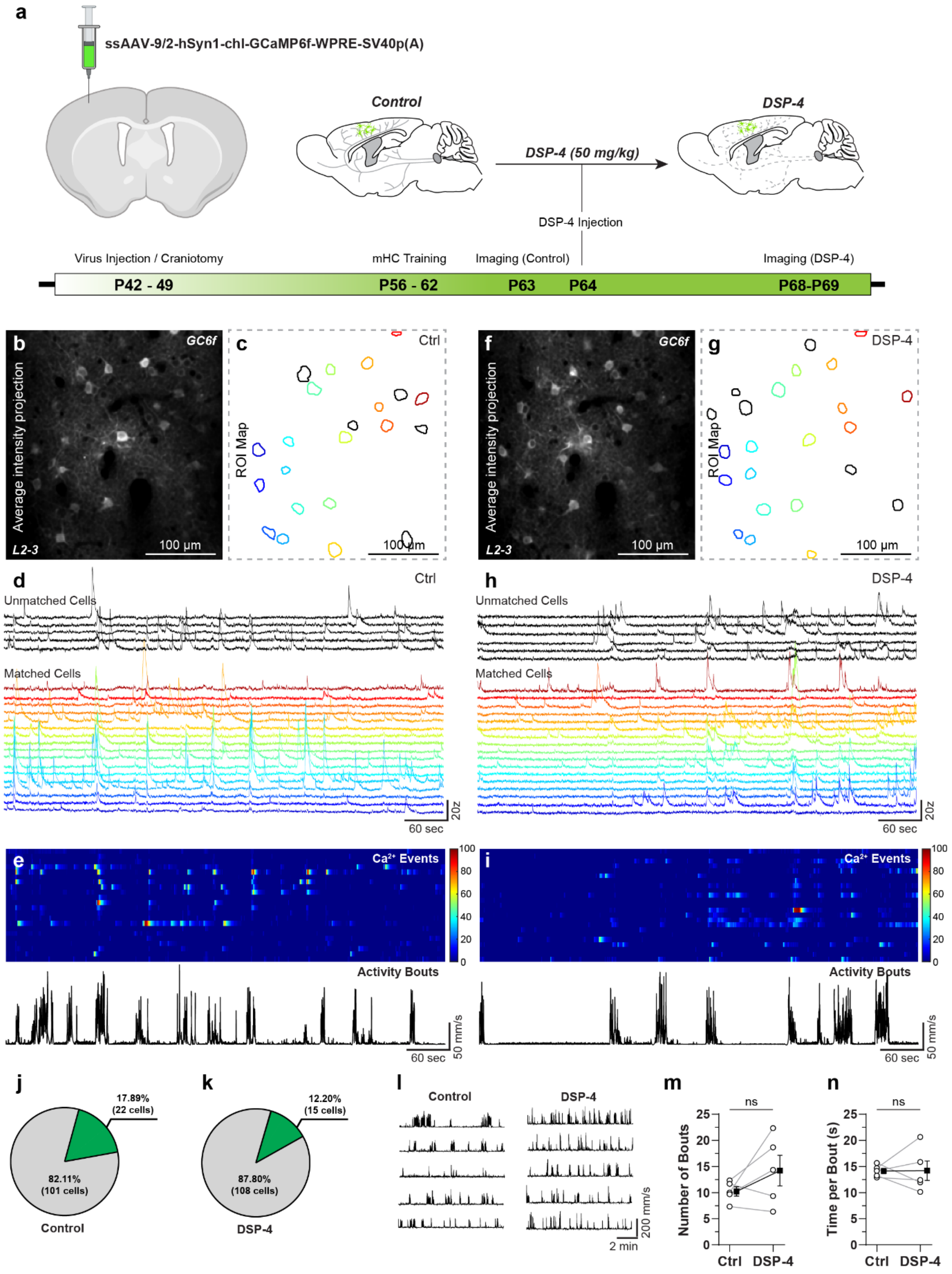

Supplementary Figure 6. Ca^{2+} activity in S1 cortical neurons (layer 2/3) following the depletion of adrenergic fibers by DSP-4.

(**a**) Schematic representation of the intracortical injection of the ssAAV-9/2-hSyn1-chI-GCaMP6f-WPRE-SV40p virus and the experimental timeline. (**b-c**) Average intensity time-

series projection of GCaMP6f-expressing neurons in S1 cortex (**b**) and its associated CaM map (**c**). (**d**) Color-coded intensity vs time Ca^{2+} traces from all the cells present in the above cortical area in the control condition. Colored traces represent cells that were imaged in both control and DSP-4 conditions. (**e**) Heat-map coded raster plots showing intensity and temporal distribution of Ca^{2+} events aligned with the associated mouse activity trace. (**f**-**g**) Average intensity time-series projection of GCaMP6f-expressing neurons in the same area of the S1 cortex after injection of DSP-4 (**f**) and its associated CaM map (**g**). (**h**) Color-coded intensity vs time Ca^{2+} traces from all the cells present in the above cortical area in the DSP-4 condition. Colored traces (Matched cells) represent cells that were imaged in both control and DSP-4 conditions. (**j**-**k**) Pie charts depicting the percentage of neurons exhibiting no change (grey) and increased Ca^{2+} activity (green) during bouts of locomotion in the control (**j**) and DSP-4 (**k**) conditions. Only matched neurons (i.e., neurons that were imaged in both control and DSP-4 conditions) are shown. (**l**) Traces showing 10-minute locomotion activity trace for mice before (left) and after (right) DSP-4 injection. (**m**-**n**) Graphs comparing the number of active bouts of locomotion (**m**), and the average duration of each bout (**n**) during a trial period of 10 minutes. Each point on the plots represents average of 2-4 10-minute trials for each mouse. All data are presented as mean ± SEM. (**m**-**n**) N = 5 mice. Wilcoxon matched-pairs rank test: $P > 0.05$). ns, not significant. s, second. z, z-score. Part of the illustration in (**a**) was created using BioRender. Source data are provided in the Source Data file.

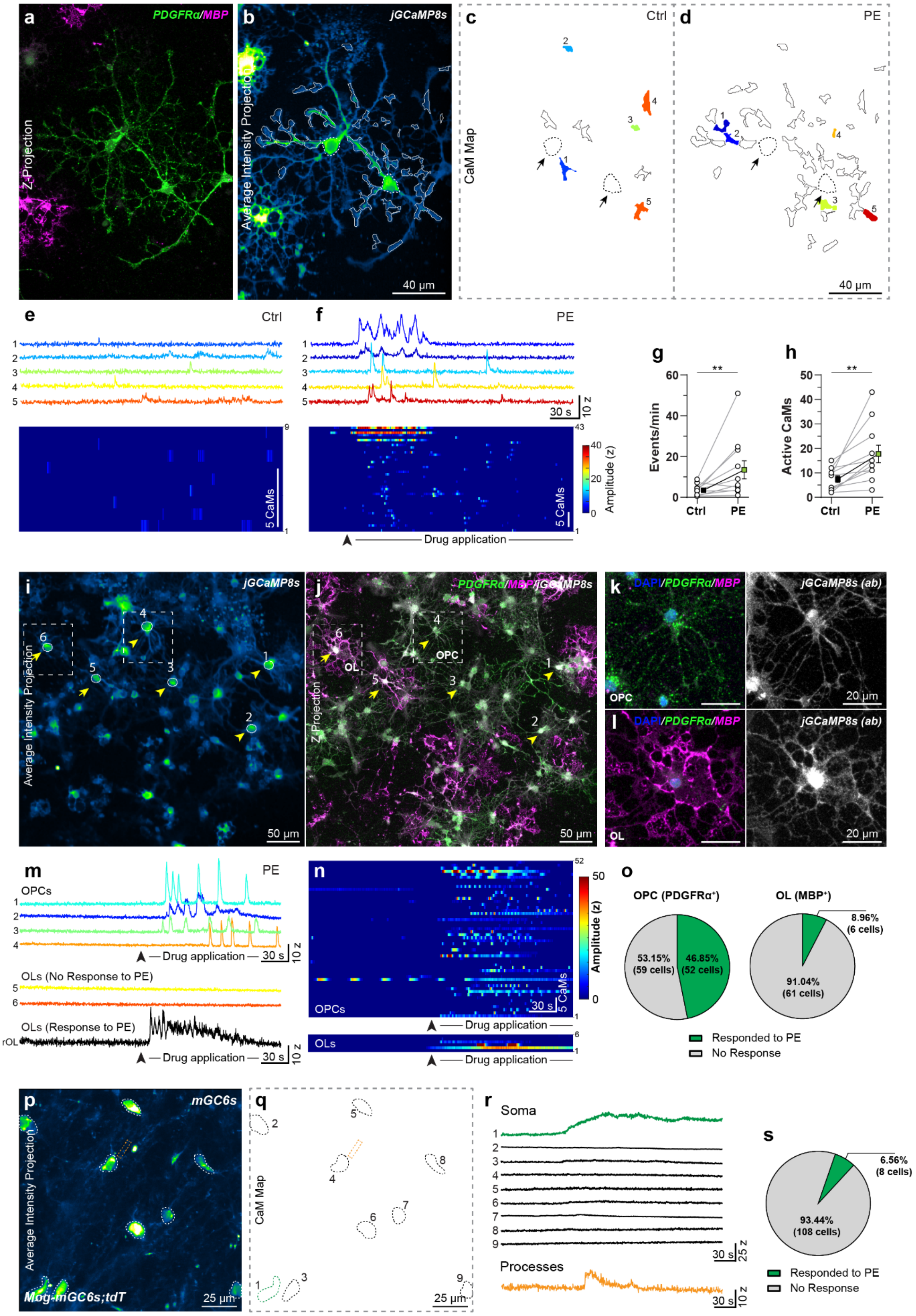

Supplementary Figure 7. OPCs in culture exhibit microdomain Ca^{2+} transients, and direct activation of α1 adrenergic receptors induces Ca^{2+} transients.

(**a**) Maximum intensity projection of a typical OPCs culture cover slip stained with PDGFRα (green) and MBP (magenta). (**b**) Average intensity projection of the cells (shown in a) expressing jGCaMP8s, overlaid with their associated CaM map. (**c**-**d**) Map of the active CaMs during control condition (**c**) and the bath application of PE (**d**). Arrows indicate the soma of the cells. (**e**-**f**) Intensity vs time Ca^{2+} traces in Ctrl (**e**, top) and during PE application (**f**, top) corresponding to colors in **c, d**, with heat-maps of the intensity and temporal distribution of Ca^{2+} events (**e**, **f**). (**g**, **h**) Graphs showing the frequency of Ca^{2+} events (**g**) and the number of active CaMs (**h**) during Ctrl and PE application. (**i**) Average intensity projection of OLCs expressing jGCaMP8s. (**j**) Confocal z-stack of the area shown in **i**, stained with PDGFRα (green), MBP (magenta) and jGCaMP8s (grey) after Ca^{2+} imaging experiments. Arrowheads indicate the soma of labeled cells, for which Ca^{2+} traces are shown in **m**. (**k**, **l**) Zoom-in images of the boxed areas shown in **j**. (**m**) Ca^{2+} activity traces of 4 OPCs (top) and 2 OLs (center) from the area in **l**, **j** during bath application of PE. The black trace shows Ca^{2+} signals in an OL that responded to PE (bottom). (**n**) Heat map of 52 OPCs (top) and 6 OLs (bottom) that responded to the bath application of PE. (**o**) Pie chart showing the proportions of OPCs (left) and OLs (right) that responded to the bath application of PE. (**p**-**q**) Average intensity projection of mGCaMP6f-expressing mature OLs in an acute cortical brain slice (**p**) and its associated CaM map (**q**). (**r**) Intensity vs time Ca^{2+} traces from all the cells in **p-q** during the bath application of PE. The green trace represents the responsive cell and the orange trace represent an active cell process (boxed area in **p-q**). (**s**) Pie chart showing the percentage of mature OLs that exhibited Ca^{2+} activity in response to bath application of PE. All data are presented as mean ± SEM. (**g**-**h**) n = 11 cells, N = 6 cover slips. Wilcoxon matched-pairs rank test: **P = 0.0049 (**g**), Paired t-test: **P = 0.0064 (**h**). (**o**) n = 111 OPCs; n = 67 OLs, N = 9 cover slips from 3 independent cultures. Scale bars, 40 µm (a); 50 µm (I, j); 20 µm (k, l), 25 µm (p, q). s, second. z, z-score. Source data are provided in the Source Data file.

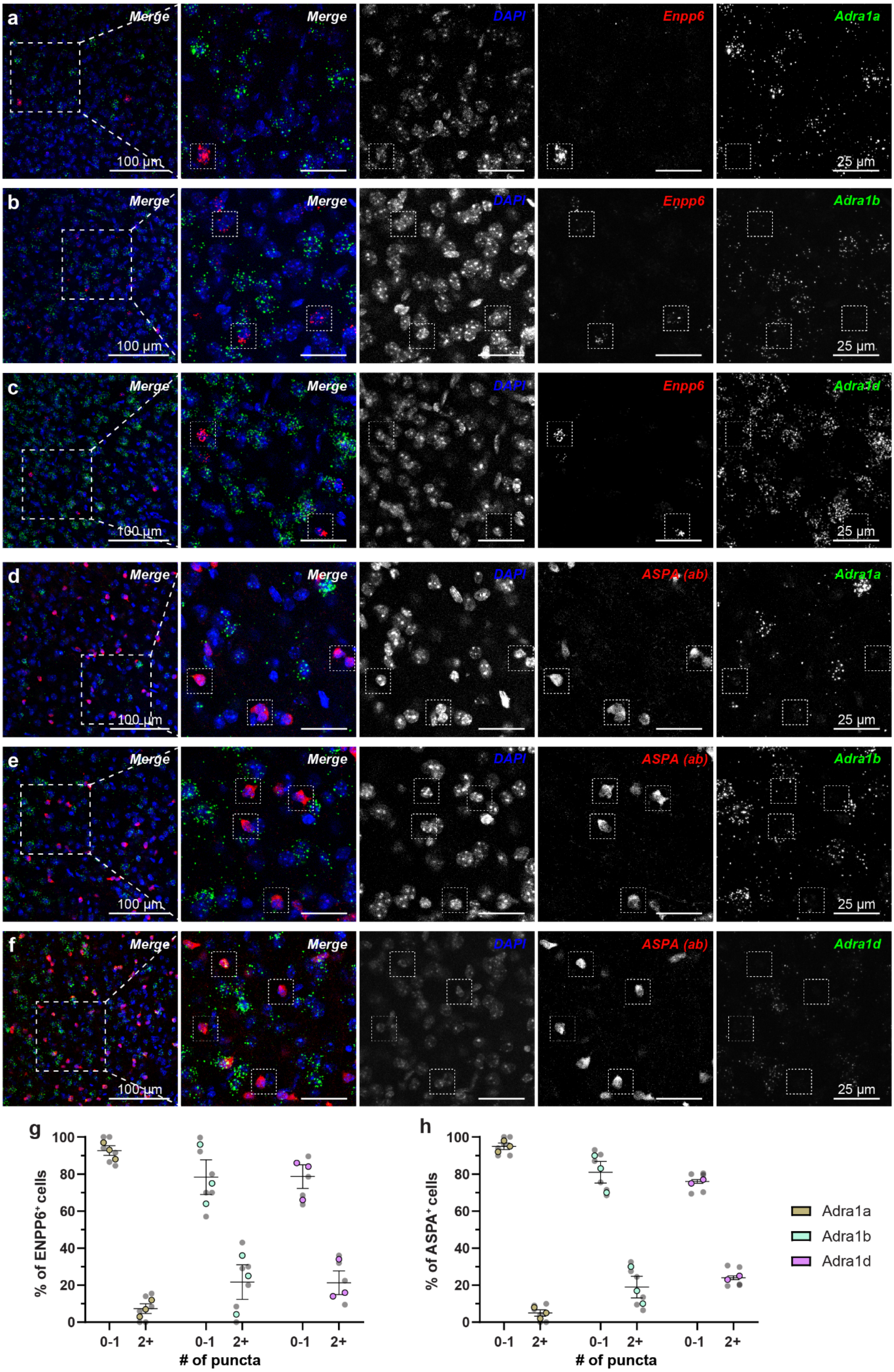

a
b
c
d
e
f
Merge
DAPI
Enpp6
ASPA (ab)
Adra1a
Adra1b
Adra1d
100 µm
25 µm
g
h
% of ENPP6⁺ cells
% of ASPA⁺ cells
100
80
60
40
20
0
0-1
2+
# of puncta
Adra1a
Adra1b
Adra1d

Supplementary Figure 8. pmOLs and OLs express low levels of α1 adrenergic receptors sub-types.

(**a**-**c**) Low (left) and high-magnification (boxed area) maximum intensity projected confocal z-stack images of cortical brain sections labeled with a pan-nuclear maker DAPI (blue), a sm-FISH probe for Enpp6 to mark pmOLs (red), and a sm-FISH probe to detect Adra1a (**a**), Adra1b (**b**) and Adra1d (**c**) (green). (**d**-**f**) Low (left) and high-magnification (boxed area) maximum intensity projected confocal z-stack images of cortical brain sections labeled with a pan-nuclear maker DAPI (blue), an anti-ASPA antibody to mark mature OLs (red), and a sm-FISH probe to detect Adra1a (**d**), Adra1b (**e**) and Adra1d (**f**) (green). (**g**) Graph showing the percentage of Enpp6^{+} cells that express Adra1a^{+} (brown), Adra1b^{+} (cyan) and Adra1d^{+} (purple) puncta. (**h**) Graph showing the percentage of ASPA^{+} cells that express Adra1a^{+} (brown), Adra1b^{+} (cyan) and Adra1d^{+} (purple) puncta. Cells with >1 puncta were considered positive for corresponding α1 adrenergic receptor sub-type expression. Data are presented as mean ± SEM. (**g**-**h**) n = 6 sections, N = 3 mice. Scale bars, 100 µm (left) and 25 µm (boxed area). Source data are provided in the Source Data file.

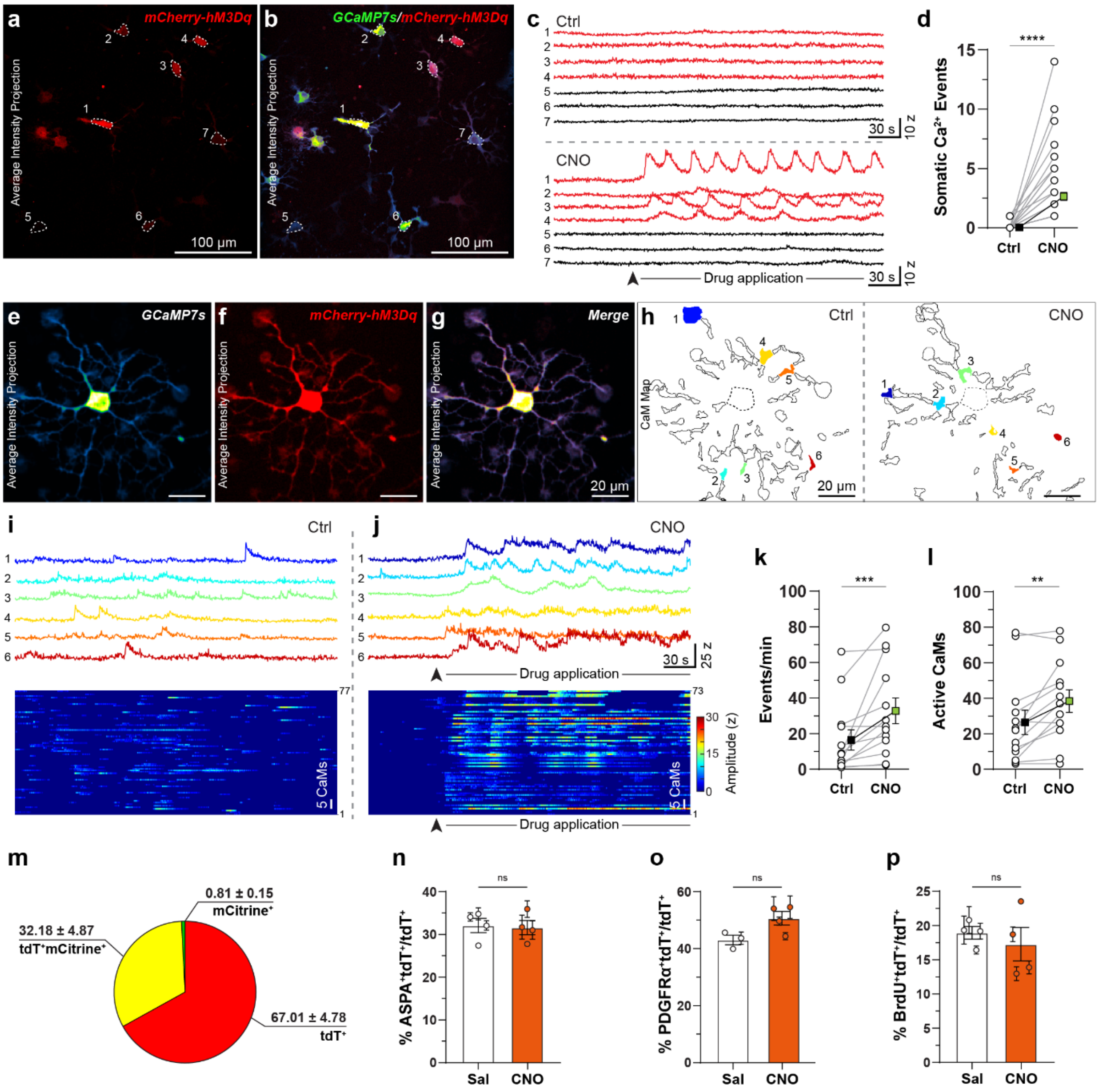

Supplementary Figure 9. hM3Dq DREADDs activation in OPCs induces Ca^{2+} transients *in vitro*.

(**a**-**b**) Average intensity time-series projection of cultured OPCs expressing mCherry-hM3Dq DREADDs (red) and GCaMP7s (green). (**c**) Intensity vs time Ca^{2+} traces from the 7 cells labelled and numbered in (**a**-**b**). Cells#1-4 (red traces, expressed mCherry-hM3Dq) responded to CNO, and #5-7 (black traces, no mCherry-hM3Dq expression) didn't respond to CNO. (**d**) Graph showing the total numbers of somatic Ca^{2+} events (in 5 min) of CNO-responsive cells before (control: Ctrl) and after bath application of CNO. (**e**-**g**) High-magnification average intensity time-series projection of a cultured OPC co-expressing GCaMP7s and mCherry-hM3Dq. (**h**) CaM map recorded before (left, Ctrl) and after bath application of CNO (right). (**i, j**) Intensity vs time Ca^{2+} traces showing Ca^{2+} signals in 6 CaMs (top) with a heat map raster plot of the intensity and temporal distribution of Ca^{2+} events in all CaMs (bottom) before (**i**) and

after bath-application of CNO (**j**). (**k-l**) Graphs showing the number of Ca^{2+} events/minute (**k**) and number of active CaMs (in 5 min) (**l**) before (Ctrl) and after bath application of CNO. (**m**) Pie chart representing the proportion of recombined cells that are expressing mCitrine only (green), tdT only (red) or both mCitrine and tdT (yellow) in the S1 cortex of *NG2-hM3Dq;tdT* mice. (**n-p**) Graphs showing the percentage of tdT+ASPA+ OLs of all tdT+ cells (**n**), tdT+PDGFRα^{+} OPCs of all tdT+ cells (**o**), and tdT+BrdU+ proliferating OPCs of all tdT+ cells (**p**) in the S1 cortex of *NG2-tdT* mice after or CNO treatment. All data are presented as mean ± SEM. (**d**) n = 63 cells from N = 18 coverslips from 5 independent cultures. Wilcoxon matched-pairs signed rank test, ****P < 0.0001. (**k-l**) n = 13 cells from N = 8 coverslips from 5 independent cultures. Wilcoxon matched-pairs signed rank test, ***P = 0.0005 (**k**). Paired t-test, **P = 0.0040 (**l**). (**o**) n = 6-8 animals. (**n-p**) n = 3-4 animals. Mann-Whitney test, P > 0.05. Scale bars 100 μm (a,b) and 20 μm (e-g). Source data are provided in the Source Data file.

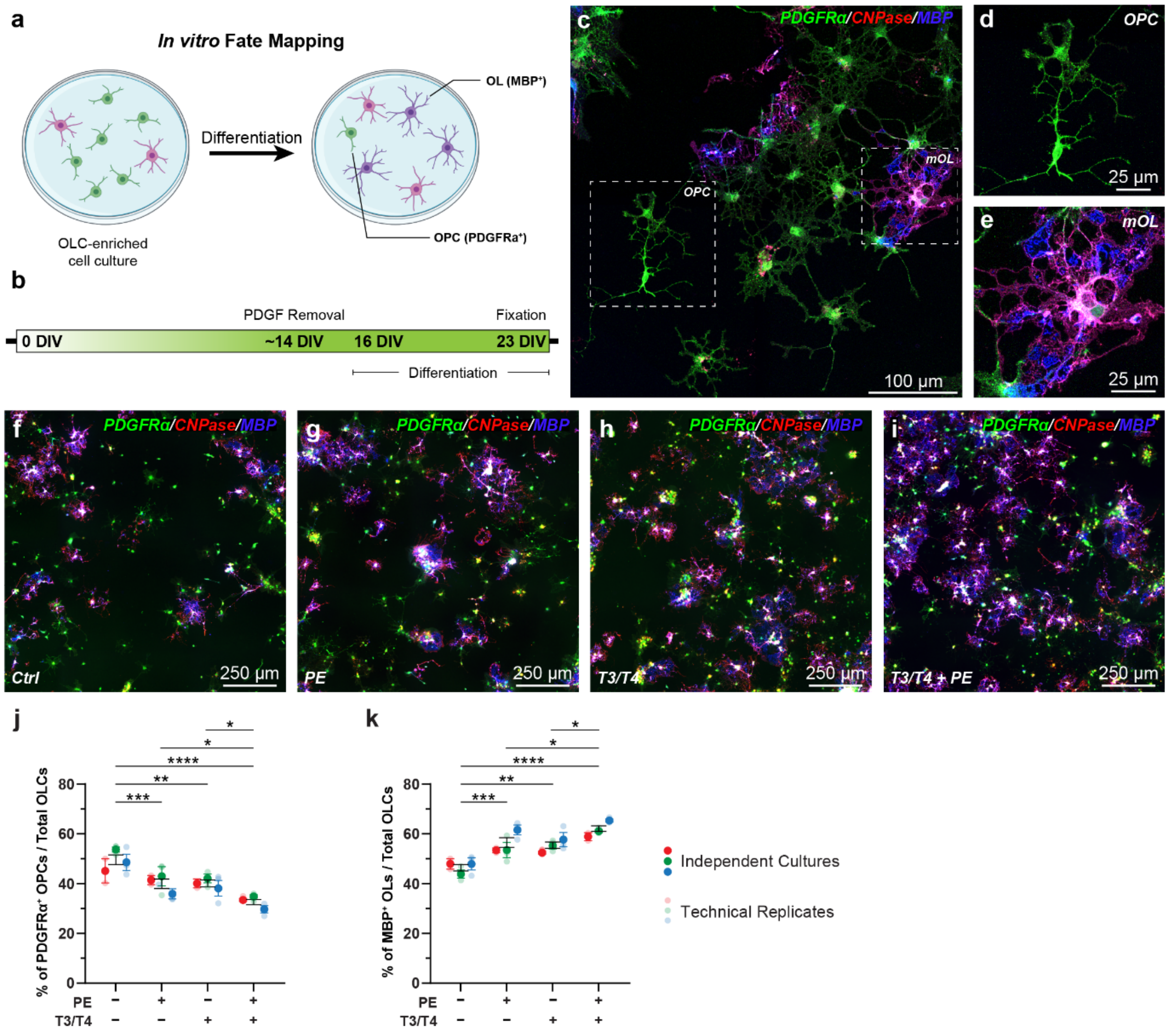

Supplementary Figure 10. Activation of α1 adrenergic receptor signaling promotes differentiation of OPCs in OLs.

(**a**) Schematic representation of a cell-fate mapping in vitro. (**b**) Experimental timeline for the differentiation and fixation of OPC cultures. (**c**) Confocal microscopy image of a typical OPC culture cover slip stained with PDGFRα (green), CNPase (red) and MBP (blue). (**d-e**) Zoom-in images of the boxed areas in **c**, showing the distinct morphology of OPCs (**d**) and OLs (**e**) in culture (**f-i**) Confocal microscopy images of OPC cultures with and without differentiation factors: Control (no factor added) (**f**), 10 μM PE (**g**), 40 ng/mL T3 + 40 ng/mL T4 (**h**), and 10 μM PE + 40 ng/mL T3 + 40 ng/mL T4 (**i**). (**j-k**) Scatter plots showing the proportion of PDGFRα^{+} OPCs (**j**) and MBP^{+} OL (**k**) with respect to the total number of oligodendrocyte lineage cells under distinct differentiation conditions. Light color small dots represent individual coverslips, and bright color larger dots represent independent cultures. All data are presented as mean ± SEM. (**j-k**) n = 8 cover slips from N = 3 independent cultures. One-way ANOVA with Tukey's multiple comparisons test: F = 18.93 (**j**) and F = 20.88 (**k**). *P < 0.05, **P < 0.01, ***P < 0.001,

****P < 0.0001. Scale bars 100 μm (c), 25 μm (d, e), 250 μm (f-i). Part of the illustration in (**a**) was created using BioRender. Source data are provided in the Source Data file.

Supplementary Methods

Supplementary Table 1 – PCR Primers

Primer Name	Mouse Line	Sequence
iCre-s	Dbh-Cre	5’-CTGCCAGGGACATGGCCAGG-3’
iCre-as	Dbh-Cre	5’-GCACAGTCGAGGCTGATCAGC-3’
R26-LSL-s	hM3Dq-Citrine	5’-CGAAGTTATTAGGTCCCTCGAC-3’
R26-hM3Dq-as	hM3Dq-Citrine	5’-GATGTTGCCGATGATGGTCAC-3’
R26-U1-s	NG2-CreER, Mogi-Cre, tdTomato	5'-CTCTGCTGCCTCCTGGCTTCT-3'
R26-U2-as	NG2-CreER, Mogi-Cre, tdTomato	5'-CGAGGCGGATCACAAGCAATA -3'
R26-U3-as	NG2-CreER, Mogi-Cre, tdTomato	5'-TCAATGGGCGGGGGTCGTT-3'
Cre-s	NG2-CreER, Mogi-Cre	5'-TGCCACGACCAAGTGACAGCAATG-3'
Cre-as	NG2-CreER, Mogi-Cre	5'- ACCAGAGACGGAAATCCATCGCTC -3'
Igs7v2-s	GCaMP6f	5'-TGCCTCCAGATCGCTAGAAT-3'
Igs7v2-as	GCaMP6f	5'-AGCTTGGCCTTCACAAAGAA-3'
loxP-s	GCaMP6f	5'-AAGTTATCACGCGCCATTTG-3'
GCaM6f-as	GCaMP6f	5'-GGGGTGTTCTGCTGGTAGTG-3'

Supplementary Table 2 – Primary Antibodies

**Primary Antibody**	**Species**	**Dilution**	**Company (Catalog #)**
anti-Aspartoacylase (ASPA)	Rabbit	1:1500	Biozol (GTX113389-100)
anti-BCas1	Guinea-Pig	1:500	Synaptic Systems (445004)
anti-BrdU	Rat	1:2000	Abcam (ab6326)
anti-CNPase	Guinea-Pig	1:1000	Synaptic Systems (355004)
anti-GFP	Chicken	1:4000	Aves (GFP-1020)
anti-MBP	Mouse	1:1000	BioLegend (808401)
anti-mCherry	Goat	1:5000	Sicgen (AB00040-500)
anti-Olig2	Rabbit	1:500	Millipore (AB9610)
anti-PDGFRα	Rabbit	1:1000	Cell Signalling Technology (3174S)
anti-RFP	Guinea-Pig	1:1500	Synaptic Systems (390004)
anti-Tyrosine Hydroxylase (TH)	Guinea-Pig	1:2000	Synaptic Systems (213104)

Supplementary Table 3 – Secondary Antibodies

Secondary Antibody	Species	Dye	Dilution	Company (Catalog #)
anti-Chicken	Donkey	Alexa 488	1:2000	Jackson ImmunoResearch (703-546-155)
anti-Goat	Donkey	Cy3	1:2000	Jackson ImmunoResearch (705-166-147)
anti-Goat	Donkey	Alexa 647	1:2000	Jackson ImmunoResearch (705-606-147)
anti-Guinea-Pig	Donkey	Cy3	1:2000	Jackson ImmunoResearch (706-166-148)
anti-Mouse	Donkey	Alexa 647	1:2000	Jackson ImmunoResearch (715-606-150)
anti-Rabbit	Donkey	Alexa 647	1:2000	Jackson ImmunoResearch (711-605-152)
anti-Rabbit	Donkey	Cy3	1:2000	Jackson ImmunoResearch (711-166-152)
anti-Rat	Donkey	Alexa 647	1:2000	Jackson ImmunoResearch (712-606-150)

Supplementary Table 4 – Phamacological Manipulation

Drug	Role	Medium	Working concentration	Company (Catalog #)
(S)-MCPG	Group I & II mGluR antagonist	Water	10 μM	HelloBio (HB6112)
Bicuculline	$GABA_A$ receptor antagonist	0.9% Saline	4 mg/kg	HelloBio (HB0895)
BMY 7378	Adrenergic $\alpha 1_D$ receptor antagonist	Water	10 μM	Tocris (1006)
CGP 39551	NMDA receptor antagonist	0.9% Saline	10 mg/kg	Tocris (1409)
CGP 55845	$GABA_B$ receptor antagonist	DMSO	5 μM	HelloBio (HB0960)
Chloroethyclonidine	Adrenergic $\alpha 1_B$ receptor alkylating agent	Water	30 μM	Sigma (B003)
Clozapine-N-Oxide (CNO)	DREADDs agonist	Water	10 μM	HelloBio (HB6149)
Clozapine-N-Oxide (CNO)	DREADDs agonist	0.9% Saline	1 mg/kg	HelloBio (HB6149)
CNQX	AMPA & Kainate receptor antagonist	Water	10 μM	HelloBio (HB0205)
D-AP5	NMDA receptor antagonist	Water	50 μM	HelloBio (HB0225)
Gabazine	$GABA_A$ receptor antagonist	Water	5 μM	HelloBio (HB0901)
NBQX	AMPA & Kainate receptor antagonist	0.9% Saline	10 mg/kg	HelloBio (HB0443)
Phenylephrine	Adrenergic $\alpha 1$ receptor agonist	Water	10 μM	Sigma (P6126)
RS 17053	Adrenergic $\alpha 1_A$ receptor antagonist	DMSO	40 μM	Tocris (0985)
Tetrodotoxin	Voltage-gated Na^{2+} channel blocker	Water	0.5 μM	HelloBio (HB1035)
