# Supplementary material for: Norepinephrine regulates calcium signals and fate of oligodendrocyte precursor cells in the mouse cerebral cortex
Source: Nat Commun. 2023 Dec 8;14:8122. doi: 10.1038/s41467-023-43920-w (PMC10709653; doi:10.1038/s41467-023-43920-w)
Supplement: Supplementary file 3 — Description of Additional Supplementary Files [file 41467_2023_43920_MOESM3_ESM.docx]

**Description of Additional Supplementary Files**

**Supplementary Video 1:** **OPC** **Ca^2+^ transients in the S1 cortex of a mouse engaging in explorative behavior**. Pseudocolored time-series images of GCaMP6f in a cortical OPC (top left) and its dynamic Ca^2+^ microdomain (CaM) map overlaid on the average intensity projection of the static reporter (tdTomato) (top right). (bottom) 2D representations of the position of the mouse in the MHC (left, grey circle), its speed (center), and the number of active CaMs at any given time. Images were acquired at 5.1 frames/s and displayed at 75 frames/s. Scale bar, 10 µm.

**Supplementary Video 2: Ca^2+^ transients in newly divided OPCs in the S1 cortex of a mouse engaging in explorative behavior**. (top left) Pseudocolored time-series images of GCaMP6f in splitting cortical OPCs. (top right) Dynamic Ca^2+^ microdomain map of daughter cell 1 (red, left) and daughter cell 2 (green, right) overlaid on the average intensity projection of the static reporter (tdTomato). (bottom) Graphs of the locomotion speed of the mouse in the MHC (left), and the number of simultaneously active CaMs in daughter cell 1 (red, center) and daughter cell 2 (green, right) at any given time. Images were acquired at 5.1 frames/s and displayed at 75 frames/s. Scale bar, 10 µm.

**Supplementary Video 3:** **Ca^2+^ transients in pmOL in the S1 cortex of a mouse engaging in explorative behavior**. Pseudocolored time-series images of GCaMP6f in a cortical pmOL (top left) and its dynamic Ca^2+^ microdomain (CaM) map overlaid on the average intensity projection of the static reporter (tdTomato) (top right). (bottom) 2D representations of the position of the mouse in the MHC (left, grey circle), its speed (center), and the number of simultaneously active CaMs at any given time. Images were acquired at 5.1 frames/s and displayed at 75 frames/s. Scale bar, 10 µm.

**Supplementary Video 4: Ca^2+^ transients in noradrenergic fibers in the S1 cortex of a mouse engaging in explorative behavior**. Pseudocolored time-series images of mGCaMP6s in noradrenergic fibers in cortex (top left) and its dynamic Ca^2+^ microdomain (CaM) map overlaid on the average time-series projection of mGCaMP6s signal (top right). (bottom) 2D representations of the position of the mouse in the MHC (left, grey circle), its speed (center), and the number of simultaneously active CaMs at any time. Images were acquired at 5.1 frames/s and displayed at 75 frames/s. Scale bar, 10 µm.

**Supplementary Video 5: Ca^2+^ transients in layer 2-3 cortical neurons in a mouse engaging in explorative behavior**. Pseudocolored time-series images of GCaMP6f in layer 2/3 cortical neurons (top left) and their dynamic Ca^2+^ microdomain (CaM) map overlaid on the average intensity projection of GCaMP6f (top right). (bottom) 2D representations of the position of the mouse in the MHC (left, grey circle), its speed (center), and the number of simultaneously active CaMs at any time. Images were acquired at 15 frames/s and displayed at 200 frames/s. Scale bar, 25 µm.

**Supplementary Video 6:** **Spontaneous and phenylephrine-evoked Ca^2+^ transients in a cortical OPC in an acute brain slice.** Pseudocolored time-series images of mGCaMP6s in a cortical OPC (left) with its dynamic Ca^2+^ microdomain (CaM) map overlaid on the average intensity projection of the static reporter (tdTomato) (right). Images were acquired at 3.1 frames/s and displayed at 75 frames/s. Time (0 - 277s): spontaneous Ca^2+^ events (in TTX, 0.5 µM); Time (278 - 550s): phenylephrine evoked Ca^2+^ events (TTX, 0.5 µM + PE, 10 µM). Scale bar, 10 µm.

**Supplementary Video 7:** **Spontaneous and phenylephrine-evoked Ca^2+^ transients in OPCs in culture.** Pseudocolored time-series images of jGCaMP8f in a cultured OPC (left) with its dynamic Ca^2+^ microdomain (CaM) map overlaid on the average intensity projection of the static reporter (mScarlet) (right). Images were acquired at 3.1 frames/s and displayed at 75 frames/s. Time (0 - 298s): spontaneous Ca^2+^ events; Time (299 - 590s): phenylephrine evoked Ca^2+^ events. Scale bar, 40 µm.

**Supplementary Video 8:** **Spontaneous and CNO-evoked Ca^2+^ transients in OPCs in culture.** Pseudocolored time-series images of GCaMP7s in a mCherry-hM3Dq+ cultured OPC (left) with its dynamic Ca^2+^ microdomain (CaM) map overlaid on the average intensity projection of the static reporter (mCherry) (right). Images were acquired at 3.1 frames/s and displayed at 75 frames/s. Time (0 - 296s): spontaneous Ca^2+^ events; Time (296 - 585s): CNO-evoked Ca^2+^ events. Scale bar, 20 µm.
