# Supplementary material for: Norepinephrine regulates calcium signals and fate of oligodendrocyte precursor cells in the mouse cerebral cortex
Source: Nat Commun. 2023 Dec 8;14:8122. doi: 10.1038/s41467-023-43920-w (PMC10709653; doi:10.1038/s41467-023-43920-w)
Supplement: Supplementary file 12 — Reporting Summary [file 41467_2023_43920_MOESM12_ESM.pdf]

nature portfolio

Corresponding author(s): Dr. Amit Agarwal

Last updated by author(s): Nov 14, 2023

Reporting Summary

Nature Portfolio wishes to improve the reproducibility of the work that we publish. This form provides structure for consistency and transparency in reporting. For further information on Nature Portfolio policies, see our Editorial Policies and the Editorial Policy Checklist.

Statistics

For all statistical analyses, confirm that the following items are present in the figure legend, table legend, main text, or Methods section.

n/a	Confirmed	
☐	☒	The exact sample size (*n*) for each experimental group/condition, given as a discrete number and unit of measurement
☐	☒	A statement on whether measurements were taken from distinct samples or whether the same sample was measured repeatedly
☐	☒	The statistical test(s) used AND whether they are one- or two-sided<br>*Only common tests should be described solely by name; describe more complex techniques in the Methods section.*
☒	☐	A description of all covariates tested
☐	☒	A description of any assumptions or corrections, such as tests of normality and adjustment for multiple comparisons
☐	☒	A full description of the statistical parameters including central tendency (e.g. means) or other basic estimates (e.g. regression coefficient) AND variation (e.g. standard deviation) or associated estimates of uncertainty (e.g. confidence intervals)
☒	☐	For null hypothesis testing, the test statistic (e.g. *F*, *t*, *r*) with confidence intervals, effect sizes, degrees of freedom and *P* value noted<br>*Give P values as exact values whenever suitable.*
☒	☐	For Bayesian analysis, information on the choice of priors and Markov chain Monte Carlo settings
☒	☐	For hierarchical and complex designs, identification of the appropriate level for tests and full reporting of outcomes
☒	☐	Estimates of effect sizes (e.g. Cohen's *d*, Pearson's *r*), indicating how they were calculated

Our web collection on statistics for biologists contains articles on many of the points above.

Software and code

Policy information about availability of computer code

Data collection	All intravital imaging data were acquired using a custom-built 2-photon microscope (Bergamo II, ThorLabs) fitted with a mode locked Ti:Sapphire pulsed laser (Chameleon Ultra II, Coherent) tuned at 940 nm. Data was acquired using the ThorImage (versions 3.1 & 4.1) software. Locomotion data was acquired using the Mobile HomeCage (Neurotar).<br>All immunohistological data was acquired using a Leica DM6000 epifluorescence microscope, or a Leica SP8 Confocal microscope.
Data analysis	All 2-photon imaging data were registered and pre-processed using ImageJ (Fiji). 2-photon imaging and Mobile HomeCage data were analyzed using a modified version of our MATLAB based CaSCaDe algorithm (see Methods) and a custom written code on MATLAB (2021a). Fate mapping and histological analysis were performed using ImageJ (Fiji) (Version 1.53t) and associated plugin (Cell Counter). Graphs and plots were generated using GraphPad Prism 9 (Version 9.4.1), and figures presented in the manuscript were assembled using Adobe Illustrator 2023.

For manuscripts utilizing custom algorithms or software that are central to the research but not yet described in published literature, software must be made available to editors and reviewers. We strongly encourage code deposition in a community repository (e.g. GitHub). See the Nature Portfolio guidelines for submitting code & software for further information.

Data

Policy information about availability of data

All manuscripts must include a data availability statement. This statement should provide the following information, where applicable:

- Accession codes, unique identifiers, or web links for publicly available datasets
- A description of any restrictions on data availability
- For clinical datasets or third party data, please ensure that the statement adheres to our policy

All data generated or analyzed during this study are included in this published article and its supplementary information files. Source data is available from the lead author upon reasonable request (AA, amit.agarwal@uni-heidelberg.de).

Research involving human participants, their data, or biological material

Policy information about studies with human participants or human data. See also policy information about sex, gender (identity/presentation), and sexual orientation and race, ethnicity and racism.

Reporting on sex and gender	N/A
Reporting on race, ethnicity, or other socially relevant groupings	N/A
Population characteristics	N/A
Recruitment	N/A
Ethics oversight	N/A

Note that full information on the approval of the study protocol must also be provided in the manuscript.

Field-specific reporting

Please select the one below that is the best fit for your research. If you are not sure, read the appropriate sections before making your selection.

[x] Life sciences [ ] Behavioural & social sciences [ ] Ecological, evolutionary & environmental sciences

For a reference copy of the document with all sections, see nature.com/documents/nr-reporting-summary-flat.pdf

Life sciences study design

All studies must disclose on these points even when the disclosure is negative.

Sample size	Sample sizes for each type of experiments were determined based on our previous experience with the analysis of similar datasets, and other related published studies (Paukert et al. Neuron 2014, Agarwal et al. Neuron 2017, Khawaja et al. Cell Reports 2021). The exact number of mice, cells, and brain sections used are specified in their respective sections.
Data exclusions	Some cells were excluded from the analysis of in vivo or slice physiology data based on imaging quality or issues in data pre-processing e.g., cells located next to highly dynamic blood-vessels, or with large z-focus drifts making the registration of image stacks impossible.
Replication	In this study, animals represent biological replicates. Cells, imaging sessions and brains sections represent technical replicates. The exact number of replicates for each experiment are indicated in the corresponding legends. The images shown throughout the manuscript are taken directly from the dataset they are meant to represent, and thus carry the same number of replicates as indicated in the legends of the corresponding figures. In cases where qualitative characterization of the mouse lines or other stainings were performed, the experiments were repeated a minimum of 3 times (N = 3 animals). The successive experiments were reproducible and did not vary between replicates.
Randomization	Mice were assigned to different experimental groups based solely on their genotype. Whenever possible, we included equal numbers of male and female mice in each group. Tissues and images were processed and analyzed in no particular order.
Blinding	All Ca2+ analyses were performed automatically using our MATLAB based CaSCaDe algorithm (Agarwal et al. Neuron 2017), and thus do not require any form of blinding. Fate mapping analyses were performed by an experimenter blinded to the genotype of the animals and/or the experimental conditions of the cell cultures.

Reporting for specific materials, systems and methods

We require information from authors about some types of materials, experimental systems and methods used in many studies. Here, indicate whether each material, system or method listed is relevant to your study. If you are not sure if a list item applies to your research, read the appropriate section before selecting a response.

Materials & experimental systems

n/a	Involved in the study
[ ]	[x] Antibodies
[ ]	[x] Eukaryotic cell lines
[x]	[ ] Palaeontology and archaeology
[ ]	[x] Animals and other organisms
[x]	[ ] Clinical data
[x]	[ ] Dual use research of concern
[x]	[ ] Plants

Methods

n/a	Involved in the study
[x]	[ ] ChIP-seq
[x]	[ ] Flow cytometry
[x]	[ ] MRI-based neuroimaging

Antibodies

Antibodies used	The following primary antibodies were used for immunohistochemical analysis: anti-ASPA (Biozol, GTX113389-100; 1:1500), anti-BCaS1 (Synaptic Systems, 445004; 1:500), anti-BrdU (Abcam, ab6326; 1:2000), anti-CNPase (Synaptic Systems, 355004; 1:1000), anti-GFP (Aves, GFP-1020; 1:4000), anti-MBP (BioLegend, 808401-clone SMI99; 1:1000), anti-mCherry (Sicgen, AB00040-500; 1:5000), anti-Olig2 (Millipore, AB9610; 1:500), anti-PDGFRα (Cell Signalling, 3174S; 1:1000), anti-RFP (Synaptic Systems, 390004; 1:1500), anti-TH (Synaptic Systems, 213104; 1:2000).<br>The following secondary anibodies were used for immunohistochemical analysis: Donkey anti-Chicken Alexa 488 (Jackson ImmunoResearch, 703-546-155, 1:2000), Donkey anti-Goat Cy3 (Jackson ImmunoResearch, 705-166-147, 1:2000), Donkey anti-Goat Alexa 647 (Jackson ImmunoResearch, 705-606-147, 1:2000), Donkey anti-Guinea-Pig Cy3 (Jackson ImmunoResearch, 706-166-148, 1:2000), Donkey anti-Mouse Alexa 647 (Jackson ImmunoResearch, 715-606-150, 1:2000), Donkey anti-Rabbit Cy3 (Jackson ImmunoResearch, 711-166-152, 1:2000), Donkey anti-Rabbit Alexa 647 (Jackson ImmunoResearch, 711-605-152, 1:2000) and Donkey anti-Rat Alexa 647 (Jackson ImmunoResearch, 712-606-150, 1:2000)
Validation	All antibodies used in this study are commercially available and well described in the literature. Anti-ASPA and anti-MBP were validated in DeFlitch et al. (Front. Cell. Neurosci., 2022). Anti-BCas1 was validated in Fard et al. (Sci. Transl. Med., 2017). Anti-BrdU and anti-Olig2 were validated in Khwaja et al. (Cell Reports, 2021). Anti-mCherry and anti-GFP were validated in Agarwal et al. (Neuron, 2017). Anti-PDGFRa was validated in Barriola et al. (Cells, 2020). Anti-RFP was validated in Zheng et al. (Mol. Metabolism, 2022). Anti-TH was validated in Liu et al. (Cell, 2018). For our application, we performed dilution series for all the antibodies to determine the optimal working concentration.

Eukaryotic cell lines

Policy information about cell lines and Sex and Gender in Research

Cell line source(s)	HEK 293T/17 cells were obtained from ATCC.
Authentication	None of the cell lines were authenticated.
Mycoplasma contamination	The cell lines were not tested for mycoplasma contamination.
Commonly misidentified lines<br>(See ICLAC register)	None

Animals and other research organisms

Policy information about studies involving animals; ARRIVE guidelines recommended for reporting animal research, and Sex and Gender in Research

Laboratory animals	All transgenic mice used in this study were either pure C57bl6/N or a mix strain between C57bl6/N and SV129 strains of mice. The generation and genotyping of the lines NG2-CreER (Zhu et al. Development, 2011, Jax #008538), Dbh-Cre (Parlato et al. Development, 2007), GCaMP6f (Madisen et al. Neuron, 2015), tdTomato reporter lines (Madisen et al. Nat. Neuroscience, 2010), Mogi-Cre (Hövelmeyer et al. J. Immunol., 2005) and hM3Dq-Citrine chemogenetic effector mice (Zhu et al. Genesis, 2016) have been previously described. For ex vivo experiments, adult mice aged 6–12 weeks old were used and for in vivo experiments, mice aged 8-16 weeks old were used unless otherwise described. Mice were maintained on a 12 hours light/dark cycle, at a temperature of 22 ± 2ºC and a humidity level between 50-60%. Food and water were provided ad libitum.
Wild animals	No wild animals were used in this study.
Reporting on sex	Both male and female mice were used for all experiments, and mice were randomly allocated to experimental groups.
Field-collected samples	No field-collected samples were used in this study.
Ethics oversight	Animal studies were approved by the Governmental Council Karlsruhe, Germany. All animal experiments were carried out in a strict compliance with German Animal Protection Law (TierSCHG) at the Heidelberg University, Germany.

Note that full information on the approval of the study protocol must also be provided in the manuscript.

Plants

Seed stocks	N/A
Novel plant genotypes	N/A
Authentication	N/A
